# Supplementary material for: Iterative improvement in the automatic modular design of robot swarms
Source: PeerJ Comput Sci. 2020 Dec 7;6:e322. doi: 10.7717/peerj-cs.322 (PMC7924708; doi:10.7717/peerj-cs.322)
Supplement: Supplemental Information 3 [file peerj-cs-06-322-s003.zip › argos3/doc/api/standalone/a00453_source.html]

ARGoS: plugins/robots/foot-bot/control\_interface/ci\_footbot\_turret\_encoder\_sensor.h Source File


- Main Page
- Related Pages
- Namespaces
- Classes
- Files

- File List
- File Members

# plugins/robots/foot-bot/control\_interface/ci\_footbot\_turret\_encoder\_sensor.h

Go to the documentation of this file.

```
00001 
00013 #ifndef CCI_FOOTBOT_TURRET_ENCODER_SENSOR_H
00014 #define CCI_FOOTBOT_TURRET_ENCODER_SENSOR_H
00015 
00016 namespace argos {
00017    class CCI_FootBotTurretEncoderSensor;
00018 }
00019 
00020 #include <argos3/core/control_interface/ci_sensor.h>
00021 #include <argos3/core/utility/math/vector2.h>
00022 
00023 namespace argos {
00024 
00025    class CCI_FootBotTurretEncoderSensor : virtual public CCI_Sensor {
00026 
00027    public:
00028 
00029       static const CRange<CRadians> ANGULAR_RANGE;
00030 
00031    public:
00032 
00033       virtual ~CCI_FootBotTurretEncoderSensor() {}
00034 
00035       const CRadians& GetRotation() const;
00036 
00037 #ifdef ARGOS_WITH_LUA
00038       virtual void CreateLuaState(lua_State* pt_lua_state);
00039 
00040       virtual void ReadingsToLuaState(lua_State* pt_lua_state);
00041 #endif
00042 
00043    protected:
00044 
00045       CRadians m_cRotation;
00046 
00047       friend class CCI_FootBotTurretActuator;
00048       friend class CRealFootBotTurretActuator;
00049 
00050    };
00051 
00052 }
00053 
00054 #endif
```

---

Generated on 10 Jul 2018 for ARGoS by 
 1.6.1
